# Blockchain based energy efficient multi-tasking optimistic scenario for mobile edge computing

**DOI:** 10.7717/peerj-cs.1118

**Published:** 2022-10-17

**Authors:** Jianbin Wu, Sami Ahmed Haider, Mukesh Soni, Ashima Kalra, Nabamita Deb

**Affiliations:** 1 Computer Science and Engineering Department, Zhejiang Normal University, Jinhua, Zhejiang, China; 2 Computing Department, University of Worcester, Worcester, United Kingdom; 3 Changdigarh University, Department of CSE, University Center for Research and Development, Mohali, Punjab, India; 4 ECE Department, Chandigarh Engineering College, Landran, Mohali, India; 5 Department of Information Technology, Gauhati University, Assam, India

**Keywords:** Mobile edge computing, Energy balance, Greedy algorithm, Blockchain, Task offloading

## Abstract

Mobile edge computational power faces the difficulty of balancing the energy consumption of many devices and workloads as science and technology advance. Most related research focuses on exploiting edge server computing performance to reduce mobile device energy consumption and task execution time during task processing. Existing research, however, shows that there is no adequate answer to the energy consumption balances between multi-device and multitasking. The present edge computing system model has been updated to address this energy consumption balance problem. We present a blockchain-based analytical method for the energy utilization balance optimization problem of multi-mobile devices and multitasking and an optimistic scenario on this foundation. An investigation of the corresponding approximation ratio is performed. Compared to the total energy demand optimization method and the random algorithm, many simulation studies have been carried out. Compared to the random process, the testing findings demonstrate that the suggested greedy algorithm can improve average performance by 66.59 percent in terms of energy balance. Furthermore, when the minimum transmission power of the mobile device is between five and six dBm, the greedy algorithm nearly achieves the best solution when compared to the brute force technique under the classical task topology.

## Introduction

With advancements in science and technology, time-sensitive applications such as augmented reality, virtual reality, and real-time gaming have exploded in popularity in recent years. The creation of such apps often necessitates the use of high-performance machine assistance. However, as mobile devices become more widespread, users are more likely to utilise them to do a variety of jobs. When a user wants to do many tasks on a mobile device, including time-sensitive apps, the device’s computational power and battery life must be adequate. However, a mobile device’s volume is frequently limited owing to its mobility, and its computational power and durability cannot be guaranteed. To address this issue, the European Telecommunications Standards Institute (ETSI) proposed mobile edge computing (MEC). MEC has advanced quickly in recent years, and it now has critical applications in video content transmission, autonomous automobiles, and other industries.

Furthermore, data sharing on the Internet has expanded due to the fast growth of the Internet of Things and fifth-generation communication technologies. According to a Cisco white paper, the number of mobile devices *per capita* will reach 1.5 by 2022, with mobile devices accounting for 20% of worldwide IP traffic (De Nitto Personè & Grassi, 2019). When all of the data created by these mobile devices is delivered to and finished by the central cloud server, network congestion is inevitable, however its use MEC to overcome these issues (Reiter, Prünster & Zefferer, 2017; Li et al., 2016).

By supporting mobile devices with their duties, MEC can lower their energy usage and time to execute these activities. However, owing to the restricted processing capacity of mobile devices, improving their computational capability while preserving energy has become a difficulty (Wei et al., 2017). As a result, most current research focuses on employing edge servers or distant clouds to boost mobile device processing capability (Li & Wang, 2018; Ren et al., 2018), such as using edge servers and cloud servers to reduce overall energy consumption (Subramanya et al., 2017; Muniswamaiah, Agerwala & Tappert, 2021; Dolui & Datta, 2017; Li et al., 2018) or task completion time (Li et al., 2018; Kim & Hong, 2019). However, the majority of current investigations ignore the issue of balancing mobile device energy consumption in the context of computing job offloading in the presence of many mobile devices (Zeng et al., 2020; Kim et al., 2020; Marjanović, Antonić & Žarko, 2018; Takahashi, Tanaka & Kawamura, 2015).

The goal of multi-device multi-energy tasking’s balancing challenge is to reduce the maximum energy usage of numerous devices. Consider the following scenario: e is an edge server, and a and b is two mobile devices. For the sake of simplicity, both mobile devices a and b are supposed to have two jobs that must be done within two time periods. Consider that edge servers and mobile devices can only conduct one task each period to simplify the description. Furthermore, the energy consumption cost is reduced when the job is given to the edge server for execution. Assume, however, that e is given both subtasks in a. In such instance, b can no longer access the edge server, causing device b’s energy consumption to be much greater than that of device a, resulting in an imbalanced energy consumption. As a result, in order to attain energy balance, the work scheduling method must be modified (Badri et al., 2018; Hung, Hsieh & Wang, 2017; Li et al., 2021; Dolui & Datta, 2017; Abbas et al., 2018; Zhao & Yang, 2021; Li et al., 2021).

Zeng et al. (2020) and Kim et al. (2020) are thought to be the most comparable to this research. The literary structure (Zeng et al., 2020) is similarly made up of three layers. It takes into account task dependencies, but its purpose is to reduce overall energy consumption rather than the balance of mobile device energy usage. The device, the edge, and the cloud are the three components of this article, and the usage of relay devices to improve the system is not explored. Multiple original devices and a wireless access point (AP) are merged by an edge server in the literature framework (Kim et al., 2020). Under two users, the study minimises the weighted total of energy use. However, this document does not employ relay devices, and the jobs in this document may be split arbitrarily, with no regard for task interdependence.

The majority of related research focuses on making use of edge server computing performance to cut down on mobile device energy utilization and task processing time. However, existing research demonstrates that the energy consumption balances between using many devices and multitasking are intractable. This energy consumption balancing issue has been addressed by updating the model for the current edge computing system. Based on this, we provide a blockchain-based analytical solution for the energy usage balance optimization problem of many mobile devices and multitasking, along with an optimistic scenario.

As a result, based on the aforementioned articles, this study improves the current work by removing the cloud server with higher energy consumption from the original structure and adding the relay device node as the task’s transit node, resulting in a mobile device. Machines and edge servers make into a three-tier structure. The following are the contributions of this article:

1. A multi-device and multi-task energy balancing greedy algorithm (GA) MMG (min-max greedy algorithm) that meets task dependence is created based on the above structure. The MMG method outperforms current minimal energy consumption algorithms while solving the multi-device multi-task energy balance issue with task dependencies.
2. The energy balancing problem is recast as a problem of minimising maximum energy consumption, and the total energy consumption optimization method and the random algorithm are compared to the MMG algorithm.
3. A significant number of comparison tests were conducted to prove the superiority of the MMG algorithm. The testing findings reveal that the MMG algorithm’s average performance in terms of energy balance may be enhanced by 66.59 percent.

## Related work

In the research of MEC, most of the teams focus on how to make full use of server resources to improve the performance of mobile devices. The goals generally fall into two categories: saving energy costs and reducing task completion time.

In terms of saving energy costs, Marjanović, Antonić & Žarko (2018) designed a dynamic computing offloading algorithm and a corresponding online offloading algorithm to solve the emotional computing offloading problem and keep the energy consumption of mobile devices. Takahashi, Tanaka & Kawamura (2015) proposed a scheduling algorithm to allocate wireless bandwidth to minimize the total cost for all users. Badri et al. (2018) offers two offloading strategies to reduce energy consumption under delay constraints. Hung, Hsieh & Wang (2017) proposed edge cloud architecture and designed a greedy algorithm to minimize the total energy consumption of mobile devices. Li et al. (2021) developed a distributed algorithm that combines “0-1” programming and coalition games and minimizes the total energy consumption of mobile devices by sharing computing results among mobile users. Samanta & Li (2018) studies the problem of reducing the total execution cost of an application under time constraints in an architecture consisting of a remote cloud and heterogeneous local processors. Abbas et al. (2018) proposed a distributed linear relaxation heuristic and a greedy heuristic to minimize the total energy consumption of mobile users. Zhao & Yang (2021) considers the energy consumption problem and the scheduling strategy of sensitive tasks and designs a distributed dynamic offloading and resource scheduling strategy. The goal is to minimize the energy consumption of mobile devices, but the energy consumption between devices is not considered—an equilibrium problem. None of those mentioned above articles has fully considered the task completion time, nor have they thought of the cooperative role of relay devices to optimize the energy consumption of mobile devices better.

To reduce the task completion time, Li et al. (2021) proposes an efficient one-dimensional search algorithm by using the Markov decision method to minimize the average delay of each computing task. Guo et al. (2020) designed a centralized, distributed, and greedy maximum scheduling algorithm on the multi-user multi-task problem to solve the multi-user multitask computing offload problem. Still, the article did not consider the task dependencies. Jiang, Li & Wu (2019) proposed a “dependency-aware” offloading scheme for “edge-cloud” collaboration and designed two algorithms to minimize the task completion time of the device under task dependency constraints and a given budget. Asheralieva & Niyato (2021) assumes that the resources of the edge server are infinite, and researches the problem of minimizing the total delay of mobile users under any given computing offloading strategy under the heterogeneous delay-sensitive computing task environment where different mobile users arrive randomly. The problem is modeled as a dynamic priority queue, and a priority transmission scheduling strategy is designed to solve it. However, none of the above articles has considered the use of relay devices (Guo et al., 2020; Jiang, Li & Wu, 2019; Asheralieva & Niyato, 2021; Wu et al., 2021).

In addition to energy consumption and task completion time, some research is devoted to solving other intractable problems encountered by edge computing. Wu et al. (2021) designs a distributed computing offload algorithm using game theory and studies the multi-user offload problem in the multi-channel wireless interference environment. Queralta et al. (2020) uses evolutionary game strategy to study the problem of multi-user computing offloading in dynamic environments. Finally, Xu et al. (2020), Huang et al. (2022), and Hou et al. (2020) exploits the mobility of UAVs to solve the low mobility problem of edge servers (Queralta et al., 2020; Xu et al., 2020; Huang et al., 2022; Hou et al., 2020).

## Consensus mechanism for block chain

The blockchain is a decentralised storage system with no administrators, in which each node owns all data. Due to its unique trust building method, blockchain is widely employed in the global deployment of the Internet of Vehicles (Reiter, Prünster & Zefferer, 2017), Internet of Things (Li et al., 2016), financial services (Wei et al., 2017; Li & Wang, 2018), smart grid (Ren et al., 2018) and other industries as a new computing paradigm and Collaboration mode (De Nitto Personè & Grassi, 2019). Several key directions for the development of the contemporary burgeoning digital sector include blockchain (Dolui & Datta, 2017), big data (Li et al., 2018), artificial intelligence (Kim & Hong, 2019), cloud computing, and network security.

The consensus mechanism refers to making nodes agree on the content in the distributed database in the process of dynamic transactions. The blockchain uses the consensus mechanism to make nodes reach a consensus on transactions, thereby weakening the function of the centralized supervision system. From the initial proof of work (PoW), practical Byzantine fault tolerance (PBFT), proof of stake (PoS) to later delegated proof of stake (DPoS), a series of consensus mechanisms such as proof of authority (PoA) (Marjanović, Antonić & Žarko, 2018). The consensus mechanism has been continuously improved, and it has evolved in different directions corresponding to additional field requirements. However, as the core technology of the blockchain, the consensus mechanism can effectively reach a consensus on the data of each node in the blockchain, complete the transaction data processing quickly, and ensure the consistency and reliability of the data. Its typical consensus mechanism is analyzed in detail as follows.

**(A) Proof of Work (PoW): **This work first proposed the idea of Proof of Work to increase the cost of spammers by calculating a specific mathematical problem (Takahashi, Tanaka & Kawamura, 2015). The PoW consensus mechanism was first introduced in the article in 1999, which also laid the foundation for the consensus mechanism used in Bitcoin proposed by Satoshi Nakamoto in later generations (Hou et al., 2020). Proof of work is one of the most typical algorithms in blockchain consensus mechanisms, such as the mining process in Bitcoin. “Miners” obtain a particular Bitcoin reward by continuously trying to calculate a random number N that meets the mining difficulty (Difficulty), as shown in Formula (1):

(1) $$N\left( {BlockHeader} \right)\ {\leq}\ target$$

The difficulty value belongs to a tiny part of the target value range (Target) in the 2^256^ input space, as shown in Formula (2):

(2) $$Targe = target*\displaystyle{{actualtime} \over {expectedtime}}$$

In the blockchain system, the block will dynamically adjust the difficulty of the threshold in a certain period (every 2 016 blocks, about 2 weeks), as shown in Formula (3):

(3) $$Difficulty = \displaystyle{{difficulty//target} \over {Target}}$$

when the system’s mining difficulty (target) remains unchanged, the actual mining difficulty is greater than the expected mining difficulty. The right side of the equal sign is greater than 1, the target threshold increases, and the mining difficulty decreases simultaneously. The mining difficulty is proportional to the target threshold difficulty. When the number of miners increases and the speed of block generation is significantly accelerated, the system will increase the difficulty of mining. The rate of block generation tends to be balanced (generally, a block is generated every 10 min). Li et al. (2018) proposed the use of the Byzantine fault tolerance algorithm (practical Byzantine fault tolerance, PBFT), which was applied to the digital asset platform with small throughput but a large number of events, which improved the efficiency based on the original Byzantine algorithm (Hung, Hsieh & Wang, 2017). The PBFT algorithm stipulates that at least 3f + 1 node need to be deployed in the whole network, which can tolerate up to If there are f malicious nodes, if a Byzantine failure occurs, the state of the entire system is determined by 2f + 1 nodes, that is, on the premise of ensuring system activity and security, a consensus is reached when the number of malicious nodes in the entire network is less than 1/3. However, the famous scientist Professor Eric proposed that distributed systems can only satisfy two of the three aspects of consistency, security and partition fault tolerance, and the PBFT algorithm could not fulfil the ecosystem at that time. Recently, Proof of Stake (PoS) first appeared in Peercoin. It uses the concept of “coinage” to control the amount of currency in the hands of miners and stipulates that currency holders must have a specific period. The longer, as shown in Formula (4) (Samanta & Li, 2018):

(4) $$Coin\; age\; = \; currency\; amount\; \times \; holding\; time$$

To ensure the system’s fairness, the higher the coin age of miners, the lower the difficulty of mining, which can reduce the possibility of users being attacked to a certain extent. Furthermore, the emergence of PoS has improved the phenomenon of excessive computing power consumption in PoW, and to a certain extent, has alleviated the previous inefficiency caused by the slow block generation time, increasing the throughput and speeding up the processing speed, but if the system The phenomenon of the richest man is prominent, which will cause the problem of centralization (Marjanović, Antonić & Žarko, 2018).

In Formulas (1)–(3), actual-time and expected time are the actual mining difficulty and expected mining difficulty, respectively; target and difficulty are the target threshold difficulty and mining difficulty of the system, respectively; Difficulty||Target is the actual mining setting for the system Difficulty value, the minimum is 1, and Target is the target threshold.

**(B) Authorized Proof of Stake (DPoS) (Takahashi, Tanaka & Kawamura, 2015): **DPoS is based on the form of democratic voting. Nodes elect N members to become the “delegation” in the system, and the node with more tokens has a higher probability of becoming a “representative”. The “representative” node in the group is responsible for collecting information, packaging transactions, and verifying transactions and newly produced blocks. The time slice is used to allocate time to the “representative” node to process things. If there is a malicious “representative”, the node will be revoked to block and cancel the “representative” resource.

Grid, and then select a new “representative”. The emergence of DPoS reduces the waste of computing power and electricity. Also, it improves the transaction processing speed and blocks throughput, but at the same time, it inevitably weakens the ability of the decentralized working model.

**(C) Proof of Authority (PoA): **Gavin, the founder of Ethereum, first proposed the proof-of-authority consensus mechanism in 2017. The PoA consensus mechanism is mainly used for reputation accumulation, and the validator needs to verify the user’s identity, not the currency held by the user (Zeng et al., 2020). A user who wants to verify a transaction first confirms their identity, links it to the verification performed, and stores it on the blockchain. When a transaction is verified, the validator’s identity is confirmed on-chain through some protocol. This identity is only determined by a small group of validators, increasing the efficiency and security of the consensus protocol. PoA does not require high computational costs or accumulation of large amounts of tokens, but it only works on private blockchains and consortium blockchain networks. In July of the same year, the Hyperledger community officially released Fabric 1.0. The emerging consensus mechanism broke the last impression of a proof-based consensus mechanism and formed an endorsing peer, orderers, and committing peers. Fabric consensus mechanism based on three types of nodes (Li et al., 2016). The Hyperledger consensus process contain following steps as:

**Step 1** The client (client SDK) creates a proposal and sends the proposal to the corresponding endorsement node according to the selected endorsement policy. The proposal contains information such as the user ID (ClientID) and the called chaincode function (BlockchainCoin function) and its parameters, timestamp (Timestamp) and client signature (ClientSig) (Wei et al., 2017).
**Step 2** The endorsing node 1erifies the client signature to ensure that an authenticated client sent the proposal. Then, the transaction request simulated by the endorsement node is executed according to the chain code in the proposal, and the endorsement node’s signature (sign TX endorsed) is appended to the generated execution result, that is, the endorsement process. The development of the simulation execution is a set of readset and writeset set based on the current world state (state database), and the endorsement signature and writeset set are sent to the client after endorsement.
**Step 3** The client verifies the signature of the endorsement result to ensure that it comes from a legitimate endorsement node. The client can check the validity of the endorsement node’s signature and the consistency between the read and write sets received from different endorsement nodes. The result generates a transaction and broadcasts it to the ordering nodes. The client can enforce mandatory checks later in the validation phase to help detect transaction failures earlier in the transaction flow to reduce overhead.
**Step 4** Order transactions using the Kafka schema in Hyperledger Fabric. The sorting node hands the received transactions to the Kafka cluster for sorting and reads a certain number of ordered transactions from the Kafka cluster according to specific rules, and packs them into blocks. After the ordering service signs the league, it distributes the block to submitting nodes.
**Step 5** After the submitting node receives the block, it can verify the league, mainly to confirm whether the read-write data set in the transaction is consistent with the data version of the world state. The submitting node uses the read moulded part of the read-write set to check the transaction’s validity and then writes the writing part of the read-write set of all verified transactions into the world state. At the same time, the submit node will use the write set to update the state database, that is, the ledger. In the event of validation failure, a validation block for aborted and committed transactions will be appended to the log. Then each transaction’s commit or abort status will be recorded.

## Models

This chapter will first introduce the model of the system in this article. Then, the symbols used in this article and their meanings are shown in Table 1.

**Table 1:** Symbols used in this article.

Symbol	Mobile device set
N	Subtask set
M	Relay Device Set
H	Edge server
h	The workload size of the jth subtask for the ith mobile device
X_i,j_	The uplink data rate between the ith mobile device and the kth relay and the uplink data rate between the kth relay and the edge server
S_ik_, S_k_	The data size of the jth subtask of the ith mobile device
E_i,j_	The computing time and local energy consumption of the i-th mobile device itself to process the j-th subtask
u^Local^_i,j_, F^Local^_i,j_	While transferring from the ith mobile device to the kth relay device, the jth subtask requires time and energy.
u^Send^_i,j,k_, F^Send^_i,j,k_	Time and energy used by the ith mobile device’s jth subtask to send data from the kth relay mechanism to the network edge
u^Send2^_i,j,k_, F^Send2^_i,j,k_	The time required to compute the jth subtask of the ith mobile device on e
u^edge^_i,j_	Total energy consumption of the jth subtask of the ith mobile device
F_i,j_	The time it takes for the jth subtask of the ith mobile device to complete the computation
u^g^_i,j_	Completion time for all subtasks of the ith mobile device
U_i_	The energy consumption of all subtasks of the i-th mobile device to complete the calculation

### System model

**Figure 1:**
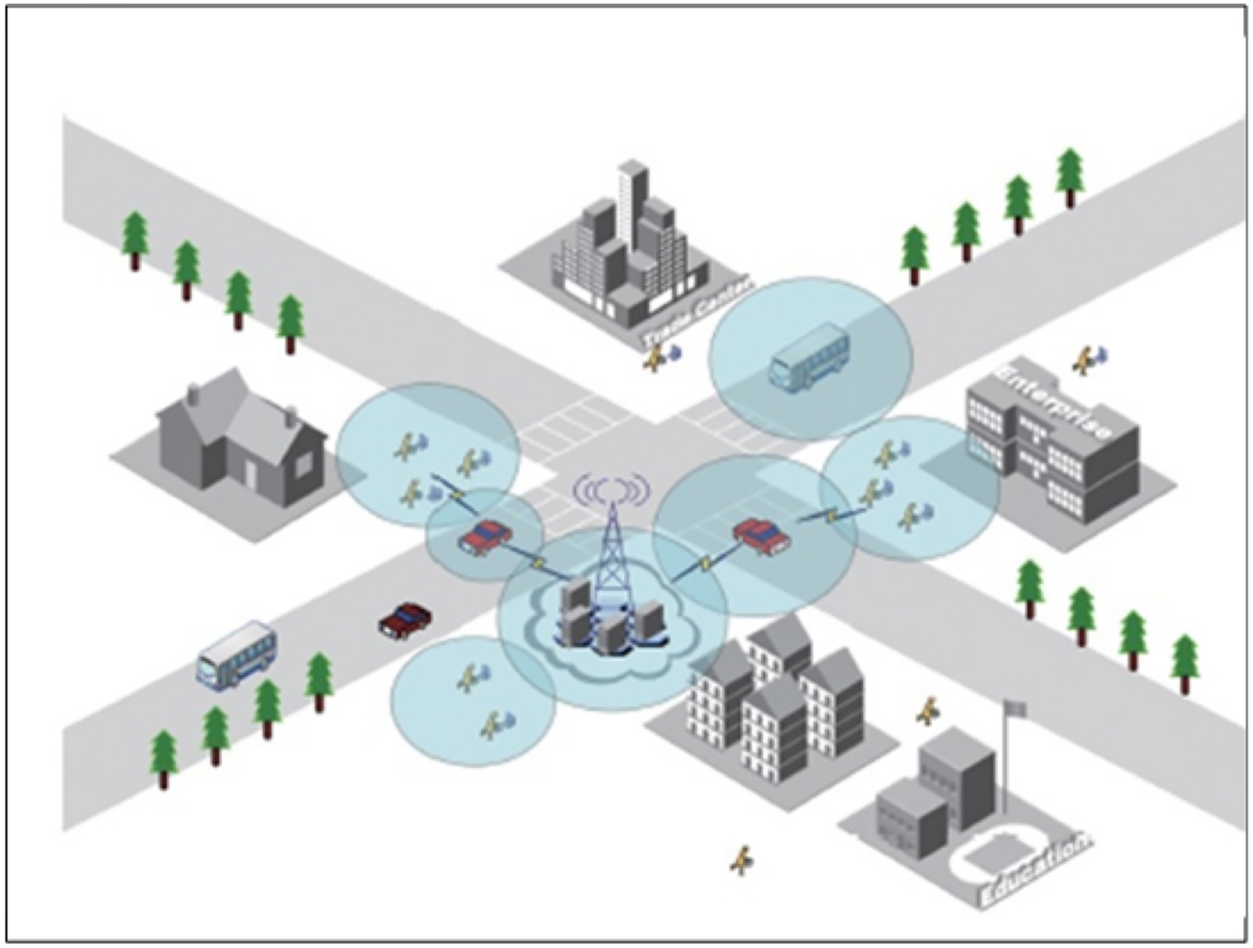
System model.

As shown in Fig. 1, the system is a mobile edge computing model with a three-tier architecture. The top layer of the system resides on an edge server with computing power, typically at a base station or remote access point. The middle layer of the system is relay devices, which are only responsible for task transmission but not task execution. The bottom layer of the system is the mobile device, each mobile device has a running application, and each application consists of multiple subtasks with task dependencies. The mobile device communicates with the relay device through a wireless connection, such as Wi-Fi. The relay device also communicates with the edge server through a wireless connection. There are multiple mobile devices in the model, represented by the set N = {n_1_, n_2_, ⋯, n_n_ }, where N is the number of mobile devices.

Furthermore, the relays in the model are represented by the set G = {g_1_, g_2_, ⋯, g_G_}, where G is the number of relays. For each mobile device, it contains M subtasks with dependencies, denoted by M = {m_1_, m_2_, ⋯, m_M_}. In the following, this article’s communication model and computing model will be introduced in detail.

### Wireless communication model

Wireless communication exists between mobile devices, relay devices, and edge servers. According to Shannon’s formula, the uplink data rate from the ith mobile device to the kth relay device can be expressed as:

(5) $${S_{i,k}} = \; {C_{i,k}}\; .Oc\; \left[ {1 + \; \displaystyle{{{Q_i}{H_{i,k}}} \over {\sigma _k^2}}} \right]$$

Similarly, the uplink data rate between the kth relay device and the edge server e is:

(6) $$S_k^{\prime} = \; C_k^{\prime}\; .Oc\; \left[ {1 + \; \displaystyle{{{Q_k^{\prime}}{H_{k,e}}} \over {{\sigma ^{{\prime}2}}}}} \right]$$

Among them, ${C_{i,k}}$ and $C_k^{\prime}$ are the channel bandwidths between the ith mobile device and the kth relay device, and between the kth relay device and the edge server e, respectively; σk and σ′ represent the kth environmental noise when the relay device and edge server e are the receivers; ${Q_i}$ and ${Q_k^{\prime}}$ are the transmission power of the ith mobile device and the transmission power of the kth relay device, respectively; ${H_{i,k}}$ and ${H_{k,e}}$ are the channel gain between the i mobile device and the kth relay device and between the kth relay device and the edge server e is calculated as:

(7) $${H_{a,b}} = {({e_{a,b}})^{ - \alpha }}$$

where ${e_{a,b}}$ is the Euclidean distance between nodes a and b, α is the path loss component, and $\rm a, b \in \{{N\cup G \cup \{e}\}\}$.

### Computational model

When j in i is executed locally on the mobile device, its execution time can be expressed as:

(8) $$u_{i,j}^{local} = \; \displaystyle{{{X_{i,j}}} \over {{d_i}}}$$

Amongst them, X (i, j) is the activity necessary to finish the j^th^ subtask in the i^th^ smart phone, d_i_ is the i^th^ mobile device’s CPU clock cycle frequency, the unit is cycle/s, and the associated power consumption is:

(9) $$F_{i,j}^{local} = {\rm \rho}_{i}\cdot {X_{i,j}}\cdot d_i^2$$

If the jth subtask in the ith smart phone is released to the kth relay device, the number of communication at this phase may be represented as: Where I is a constant that relies on the architecture of the mobile device, the number of communication at this point can be expressed as:

(10) $$u_{i,j,k}^{sen{d_1}} = \; \displaystyle{{{E_{i,j}}} \over {{S_{i,k}}}}$$

Among them, ${E_{i,j}}$ is the data size of the jth subtask of the i^th^ mobile device. The energy consumption of this process can be expressed as:

(11) $$E_{i,j,k}^{sen{d_1}} = \; {q_i}\;.\;u_{i,j,k}^{sen{d_1}}$$

If the jth subtask in the ith mobile device is offloaded from the kth relay device to the edge server e, the transmission time of this process can be expressed as:

(12) $$u_{i,j,k}^{sen{d_2}} = \; \displaystyle{{{E_{i,j}}} \over {{S_k}}}$$

The corresponding energy consumption is:

(13) $$E_{i,j,k}^{sen{d_2}} = \; q_i^{\prime}\;.\;u_{i,j,k}^{sen{d_2}}$$

The computation time to complete the jth subtask of the ith mobile device on the edge server e is:

(14) $$u_{i,j}^{edge} = \; \displaystyle{{{X_{i,j}}} \over {{d_e}}}$$

Among them, ${d_e}$ is the CPU clock cycle frequency of e, and the unit is cycle/s.

Therefore, the energy consumption for completing the jth subtask of the ith mobile device is:

(15) $${E_{i,j}} = \; E_{i,j,k}^{Local}.\left( {1 - \; \mathop \sum \nolimits_{k = 1}^G {A_{i,j,k}}} \right) + \mathop \sum \nolimits_{k = 1}^G (F_{i,j,k}^{sen{d_1}}\;.\;{A_{i,j,k}})$$

where $\mathop \sum \nolimits_{k = 1}^G {A_{i,j,k}}$ $\in$ {0,1}, ${A_{i,j,k}}$ is the assignment strategy of the jth subtask of the ith mobile device. When $\mathop \sum \nolimits_{k = 1}^G {A_{i,j,k}}$ = 0, it means that the jth subtask in the ith mobile device is offloaded from the ith mobile device to the kth relay device; when $\mathop \sum \nolimits_{k = 1}^G {A_{i,j,k}}$ = 1, it means that the jth subtask of the ith mobile device performs computation locally.

### Task dependencies

This section defines dependencies between subtasks. The completion time of the subtask is divided into the following parts.

Let T ready_1_ i,j be the time when the jth subtask of the ith mobile device is ready to be processed locally, then:

(16) $$U_{i,j}^{read{y_1}} = \; ma{x_{s \in {\pi _J}}}.\left\{ {U\; G_{i,s}^{Local}} \right\}\;$$

where ${\pi _J}$ is the set of predecessors to the jth subtask, s ∈ ${\pi _J}$. TF local, s is the local completion time of the sth subtask of the ith mobile device, which can be calculated as:

(17) $$U\; G_{i,s}^{Local} = \; u_{i,j}^{edge} + U_{i,j}^{read{y_1}}$$

Similarly, $U\; G_{i,s}^{Local}$ is the completion time of the jth subtask of the ith mobile device on edge server e. It can be represented as:

(18) $$U\; G_{i,j}^{Local} = \; u_{i,j}^{edge} + U_{i,j}^{read{y_1}}$$

where $U_{i,j}^{read{y_1}}$ j is the time when the j^th^ subtask of the ith mobile device is ready to be processed on the edge server e, which can be calculated as:

(19) $$U_{i,j}^{read{y_2}} = \; ma{x_{s \in {\pi _J}}}.\left\{ {u_{i,s}^d} \right\} + \; u_{i,j,k}^{sen{d_1}} + \; u_{i,j,k}^{sen{d_2}}$$

Let $u_{i,s}^d$ be the completion time of the j^th^ subtask of the ith mobile device, which can be calculated as:

(20) $$u_{i,s}^d = \; U\; G_{i,j}^{Local}.\left( {1 - \; \mathop \sum \nolimits_{k = 1}^G {B_{i,j}}\left( k \right)} \right) + \; U\; G_{i,j}^{edge}.\left( {1 - \; \mathop \sum \nolimits_{k = 1}^G {B_{i,j}}\left( k \right)} \right)$$

Therefore, the time required for the i^th^ mobile device to complete all subtasks is:

(21) $${U_i} = ma{x_{j \in N}}\left\{ {u_{i,s}^d} \right\}$$

To sum up, the total energy consumption of the i^th^ mobile device can be expressed as:

(22) $${F_i} = \mathop \sum \limits_{j = 1}^G {F_{i,j}}$$

### Problem definition

This section will give a formulaic definition of minimizing the maximum energy consumption. The problem of reducing the maximum energy consumption is described as follows:

(23) $${\rm Obj\!\!}:{\rm \; min}\left\{ {{\rm max}\left( {Fi} \right)} \right\}$$

(24) $${\rm U}i \leq U,\;\; \forall i \in {\rm N}$$

(25) $$ma{x_{j \in N}}\left\{ {u_{i,s}^d} \right\} \le U_{i,j}^{read{y_1}},{\rm \; }\forall i\; \in {\rm \; N},{\rm \; }\forall j\; \in {\rm \; M}$$

(26) $$ma{x_{j \in N}}\left\{ {u_{i,s}^d} \right\} \le U_{i,j}^{read{y_2}},{\rm \; }\forall i\; \in {\rm \; N},{\rm \; }\forall j\; \in {\rm \; M}$$

(27) $$\mathop \sum \limits_{k = 1}^G {A_{i,j,k}}\; .\left( {1 - \; \mathop \sum \limits_{k = 1}^G {A_{i,j,k}}} \right) = 0\; ,{\rm \; }\forall i\; \in N,\forall j\; \in {\rm \; M},\forall k\; \in {\rm \; G}$$

where U is the budget time for the given ith mobile device to complete all subtasks Eq. (24) is the constraint on the completion time of the ith mobile device. Equations (25) and (26) are the dependency constraints of subtasks, ensuring that the jth subtask of the ith mobile device can only start executing after all its predecessor subtasks are completed. Equation (27) indicates that the jth subtask of the ith mobile device can only be committed locally or at the edge server, e.

According to the existing research, the problem of minimizing the maximum energy consumption is an optimization problem of finding a scheduling strategy, which can be reduced to the minimum, full-time problem in the literature, which reduces the minimum, full-time pain to NP-complex integer programming problem. Therefore, the issue of minimizing the maximum energy consumption is NP-hard.

## Performance evaluation

In this chapter, experiments are designed to investigate the performance of the proposed algorithm. This article uses Matlab r 2016a to conduct many experiments and get the experimental results. This article sets the coverage as 50 m × 50 m (Hou et al., 2020). By default, the number of subtasks, mobile devices, and relay devices is set to 4, 3, and 3, respectively, and other parameters in the experiments (Jiang, Li & Wu, 2019; Asheralieva & Niyato, 2021; Wu et al., 2021) are shown in Table 2. In the whole experiment process, the article assumes that the data size and workload size of computing tasks follow a normal distribution in the range of (25 Kbit, 1,024 Kbit), with a mean of 512 and a standard deviation of 256. In addition, this article also designs an experimental comparison algorithm for the total energy consumption minimization algorithm (TECM).

**Table 2:** Experimental parameters.

Parameter	Numerical value	Parameter	Numerical value
alpha	0.1	Bk′/MHz	5
Bi,k/MHz	5	pk′/dBm	5
pi/dBm	5	fe/GHz	4
fi/GHz	0.8	σ′/dBm	−70
ρi	0.3 × 10^−27^	σk/dBm	−70

### Experiment 1

**Figure 2:**
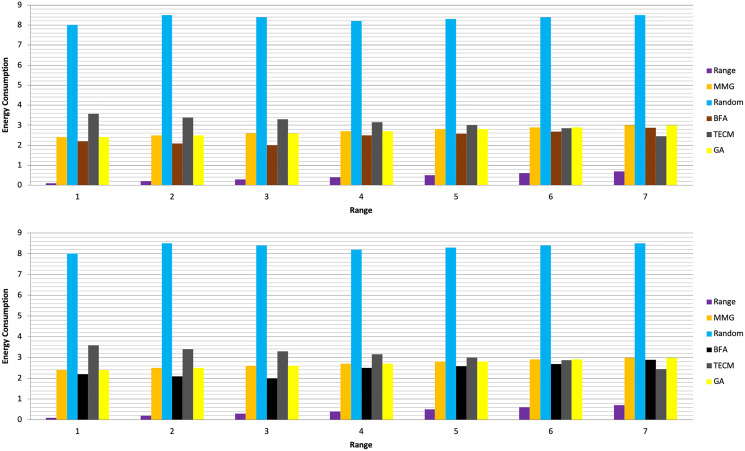
Maximum energy consumption. (A) Transmission Power = 5 dbm. (B) Transmission Power = 6 dbm.

**Figure 3:**
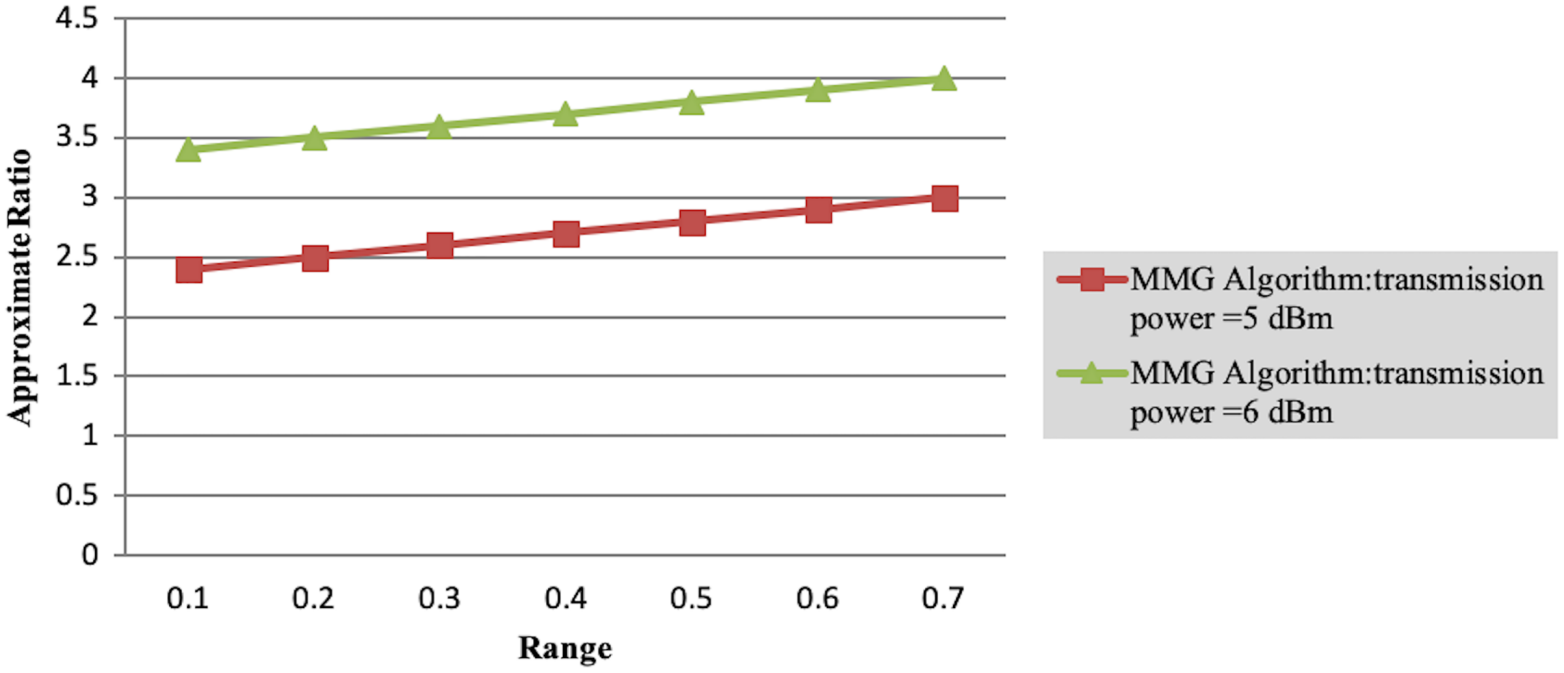
Approximation ratio of greedy algorithm.

In Experiment 1, the article studies the change of the relationship between the path loss component and the maximum energy consumption of the subtask. Figure 2 depicts that the maximum energy consumption of the subtask increases with the path loss component. From Eqs. (5)–(7), (10), (12), the maximum energy consumption of subtasks is proportional to the path loss component, which explains the shape of the curves in the experimental Fig. 2 with Table 3. Experiments show that when the transmission power of the mobile device is five dBm and six dBm, the performance of the MMG algorithm in minimizing the maximum energy consumption of subtasks is improved by 66.59% and 61.87% on average compared with the random algorithm, which is close to the maximum obtained by the brute force algorithm (BFA) optimal solution. Figure 3 is based on the results of Figs. 2A and 2B, which show that the approximate ratios of the MMG algorithm are 1.096 9 and 1.098 4 when the minimum transmit power of the mobile device is five dBm and six dBm, respectively.

**Table 3:** Evaluation of experiment 1.

Range	MMG	Random	BFA	TECM	GA	MMG	Random	Brute force	TECM	GA
	Transmission power = 5 dBm					Transmission power = 6 dBm				
0.1	2.4	8	2.2	3.58	2.4	3.4	8.5	2.5	4.58	3.4
0.2	2.5	8.5	2.1	3.39	2.5	3.5	7.5	2.67	4.39	3.5
0.3	2.6	8.4	2	3.29	2.6	3.6	7.3	2.78	4.29	3.6
0.4	2.7	8.2	2.5	3.15	2.7	3.7	7.9	2.2	4.15	3.7
0.5	2.8	8.3	2.58	3	2.8	3.8	8.6	2.296	4	3.8
0.6	2.9	8.4	2.69	2.86	2.9	3.9	8.4	2.45	3.86	3.9
0.7	3	8.5	2.88	2.45	3	4	8	2.78	3.45	4

### Experiment 2

**Figure 4:**
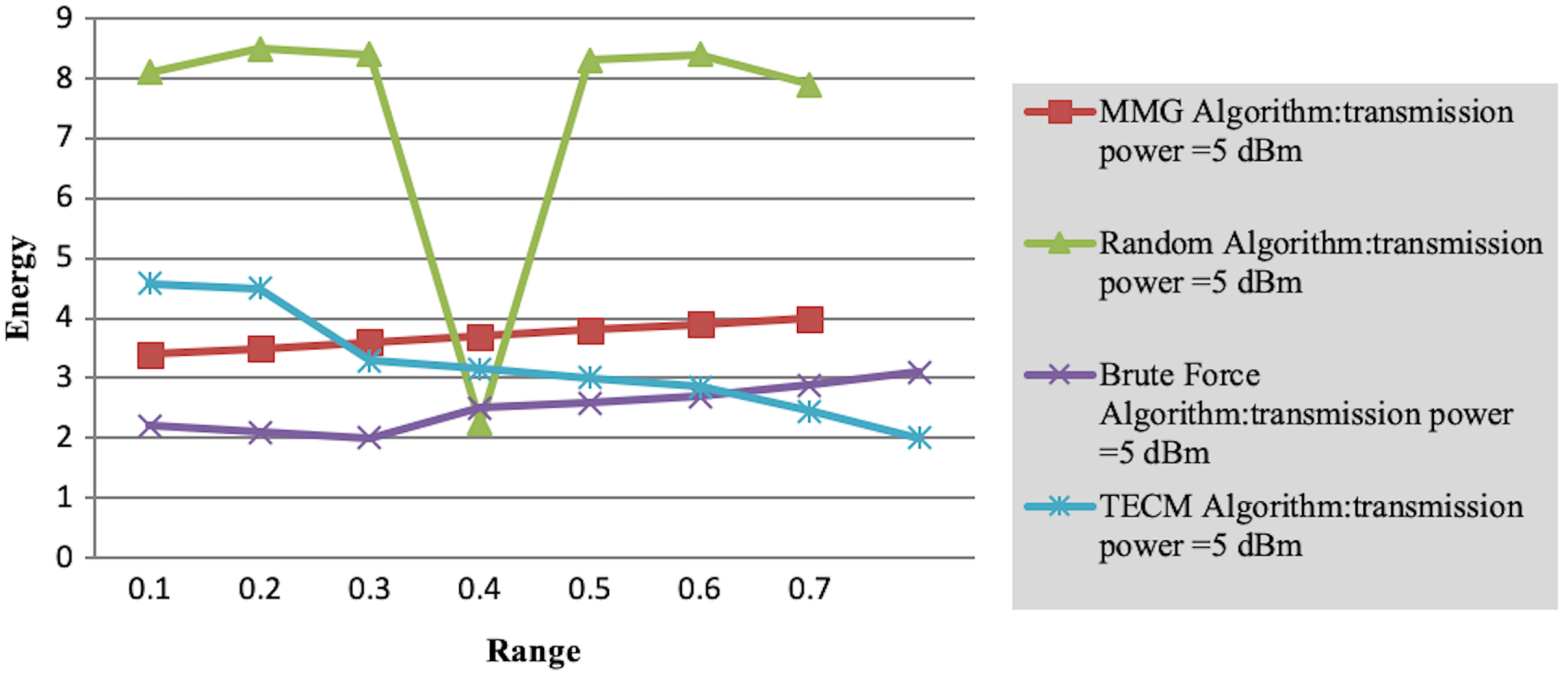
Maximum energy consumption of subtask *vs* time constraint.

Experiment 2 investigates the maximum energy consumption of subtasks under changing time constraints. In Figs. 3 and 4 the minimum transmits the power of the mobile device is five dBm. The results show that the greedy algorithm outperforms the total energy consumption optimization and random algorithms.

Finally, in the case where the path loss component is 0.1, we observe the variation of the maximum energy consumption of subtasks with the number of task layers. As shown in Table 4, as the number of tasks increases, the completion time of each subtask decreases accordingly, resulting in jobs that need to be executed in a shorter time. This leads to a rise in the energy consumption required to complete the task. However, the MMG algorithm is better than the TECM algorithm and the random algorithm in the maximum energy consumption of the minimum subtask. As shown in Table 4, as the number of subtasks increases, the leading energy consumption of subtasks increases, for the transmission power of 5 dBm, the approximate ratio of MG is 1.353 7, 1.353 8.

**Table 4:** Maximum energy consumption of subtask V.S $time$ constraint (Transmission power = 5 Dbm).

Range	MMG algorithm	Random algorithm	Brute force algorithm	TECM algorithm	Time constraint
0.1	3.4	8.1	2.2	4.58	2.4
0.2	3.5	8.5	2.1	4.49	2.5
0.3	3.6	8.4	2	3.29	2.6
0.4	3.7	2.25	2.5	3.15	2.7
0.5	3.8	8.3	2.58	3	2.8
0.6	3.9	8.4	2.69	2.86	2.9
0.7	4	7.9	2.88	2.45	3

### Experiment 3

**Figure 5:**
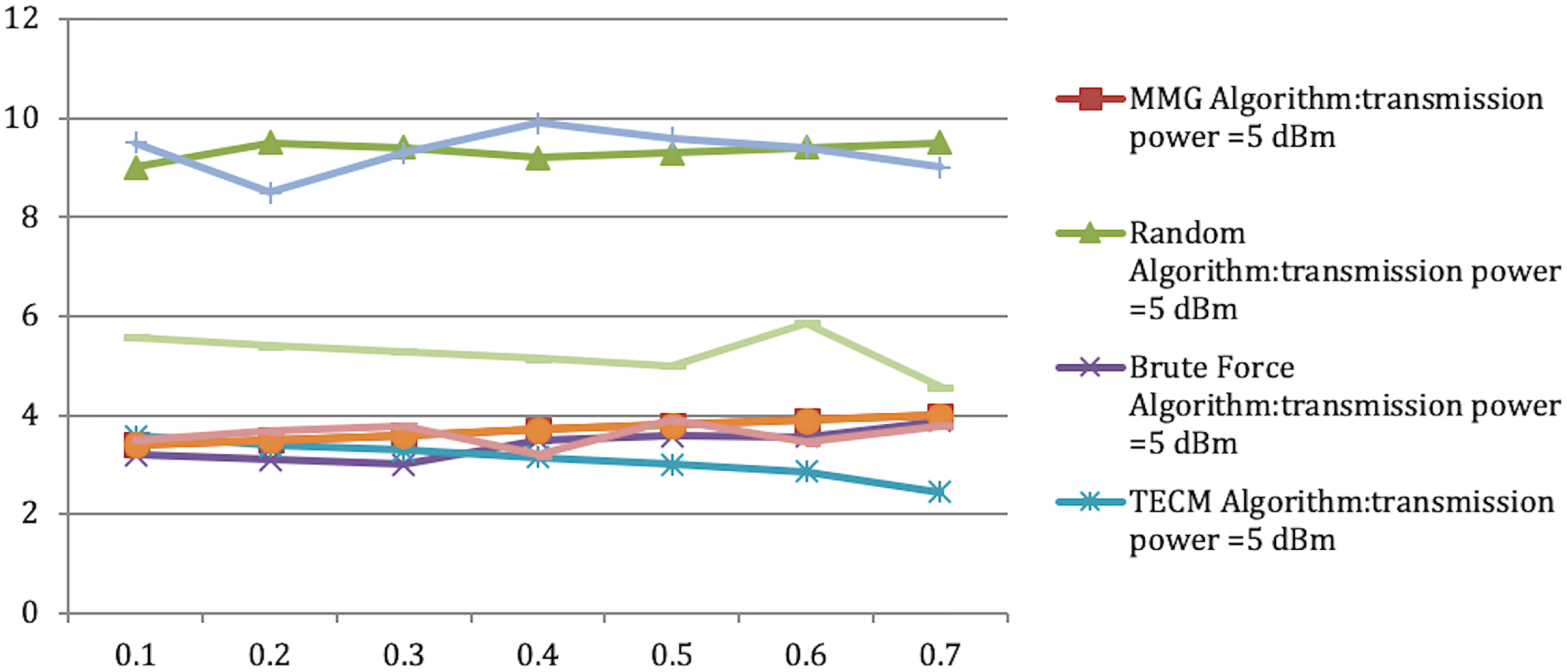
Approximation ratio of MMG algorithm.

Accomplished inside the confines of the room As a result, the amount of energy needed to execute the work increases. However, in terms of maximum energy consumption of the smallest subtask, the MMG method outperforms both the TECM and the random algorithms. As shown in Table 5 and Figure 5, the leading power consumption of subtasks rises as the quantity of subtasks grows, and when the portable device’s signal strength is five dBm and six dBm, respectively, the approximate MMG ratios are 1.353 7, 1.353 8.

**Table 5:** Maximum energy consumption of subtask V.S *the nuber of subtask*.

Range	MMG	Random	Brute force	TECM	Subtask	MMG	Random	Brute force	TECM	Subtask
	Transmission power = 5 dBm					Transmission power = 6 dBm				
0.1	3.4	9	3.2	3.58	3.4	3.4	9.5	3.5	5.58	3.4
0.2	3.5	9.5	3.1	3.39	3.5	3.5	8.5	3.67	5.39	3.5
0.3	3.6	9.4	3	3.29	3.6	3.6	9.3	3.78	5.29	3.6
0.4	3.7	9.2	3.5	3.15	3.7	3.7	9.9	3.2	5.15	3.7
0.5	3.8	9.3	3.58	3	3.8	3.8	9.6	3.9	5	3.8
0.6	3.9	9.4	3.56	2.86	3.9	3.9	9.4	3.45	5.86	3.9
0.7	4	9.5	3.88	2.45	4	4	9	3.78	4.56	4

This article provides a detailed description of the proposed MMG algorithm. This article divides U into several parts according to the number of subtasks, and each subtask of each layer should end in the interval after the division is completed. First, each subtask’s computation model is determined based on time and energy consumption conditions. Then, for each subtask, an allocation scheme is obtained by a min-max algorithm. In addition, it is then judged whether the allocation scheme satisfies the time limit.

## Validity of algorithm design and result analysis

Based on the above analysis, the energy consumption of different subtasks calculated locally and migrated to the edge server is obtained through calculation, and an energy consumption matrix is formed. Then, a relay device node is randomly assigned for each mobile device source node. Finally, assuming F_max_ is the maximum energy consumption from node a to relay b, find F_max_, set all relays to “unused”, and set to “used” when the relay device is occupied.

Get allocation policy. It is judged whether there are other relay device nodes available except the relay device nodes allocated by the maximum energy consumption. If available, replace the relay device node and determine the new F_max_ (adjust the energy consumption so that the total energy consumption obtained by the allocation strategy is as small as possible, and the energy consumption difference is more minor). If no other available relay device node is found, it starts to judge whether the allocation strategy satisfies the time constraint. If not, the process is re-allocated; the feasible allocation strategy is obtained if it is met.

**Theorem 1:** Set the number of mobile devices to be M, the number of subtasks of each mobile device to be N, and the number of relay devices to be G. The total time complexity of the algorithm is P(NMG + NMG^2^).

Proof First, the algorithm proposed in this article is divided into three levels of nested loops to calculate the time consumption and energy consumption, and its time complexity is P(NMG). Then, the time complexity of the min-max function is analyzed. The complexity of each iteration is P(G), and there are at most NG pairs per iteration, so the total min-max time complexity is P(NMG^2^). In terms of time checking, this is a sum function with only one level of loops, including two groups of nested. While loops, each while loop does not exceed H times, so its time complexity is P(NMG^2^). Also, the second step is executed under different subtask layers, so the total time complexity of the second step is O(MNH2). The whole time complexity of this algorithm is P(NMG + NMG^2^).

**Theorem 2:** The bipartite graph of any mobile edge computing structure can be transformed into an energy consumption matrix. Denote ε and N as an arbitrarily small positive constant and the number of subtasks, respectively, the approximate ratio of this greedy algorithm can reach (1 + ε)^N^.

Prove In Theorem 1, the algorithm’s time complexity is P(NMG + NMG^2^), and the maximum number of iterations is G. For a subtask, according to Formulas (11) and (13), let its transmission energy ratio be σ and ψ, respectively; according to Formula (9), let its calculated energy ratio be γ. The energy consumption of each “device-relay” pair can be expressed as ${\rm F_{i,j} = min(\sigma_{ i,j} + \gamma_j , {\psi} _{i,j} )}$. The maximum and effective minimum energy consumption of “source-relay” is set to F_max_ and F_max_, respectively. Then there are:

$\rm F_{max} \leq min(\sigma_{i,j} + \gamma_j , {\psi}_{i,j} )\cdot {\widetilde\omega}_{max}$

$\rm F_{max} \geq min(\sigma_{i,j} + \gamma_j , {\psi}_{i,j} )\cdot {\widetilde\omega}_{min}$

Therefore, the upper bound on the energy consumption of each “device-relay” pair is F_max_. Let *λ* = εF_max_/μ, where *λ* is the step size of the average adjustment of the maximum energy consumption for each “device-relay”, and μ is the maximum number of iterations, then F_max_ = *λ*μ/ε. Let F_op_(D*) be minimized for each “device-relay.”

The optimal solution for the maximum energy consumption of D* corresponds to the optimal policy. Let C′ be the maximum energy consumption of each “device-relay” pair in the greedy algorithm, then F_op_(D*) ≤ F(D′). And *λ* = εF_max_/μ, then there is *λ*μ = εF_max_. Therefore, λμ is one of the upper bounds on the energy consumption adjustment of mobile devices. Accordingly, there are:

(28) $$F{\rm op}\left( {{{\rm D}^{\rm *}}} \right) \le F\left( {D{\rm '}} \right) \le F{\rm op}\left( {{{\rm D}^{\rm *}}} \right) + \lambda \mu$$

According to Eq. (28) and 0 < F_min_ < Fop(D*), we have:

$ \eqalign{& F\left( {{\rm {D}^{\prime}}} \right) \le {\rm Fop}\left( {{{\rm D}^{\rm *}}} \right) + \lambda \mu \; = {\rm \; }F{\rm op}\left( {{{\rm D}^{\rm *}}} \right) + \epsilon F{\rm max} \cr&\quad = F{\rm op}\left( {{{\rm D}^{\rm *}}} \right) + \displaystyle{\epsilon \over {\epsilon ^{\prime}{{\rm F}_{{\rm max}}}}} \le F{\rm op}\left( {{{\rm D}^{\rm *}}} \right) + \displaystyle{\epsilon \over {\epsilon ^{\prime}F{\rm op}\left( {{{\rm D}^{\rm *}}} \right)}} } $

Let ε″ = ε/ε′, where both ε and ε′ are positive constants, and ε′ is approximately equal to ῶ_min_/ῶ_max_, then F(D′) ≤ (1 + ε″)Fop(D*).

## Conclusion

This work investigates the NP-hard issue of energy balancing in multitasking with several mobile devices. The cloud server that consumes more transmission energy is deleted from the original design. The task’s relay device node is added as the task’s transfer node, resulting in three-layer architecture of smart phones, relay devices, and edge servers. Simultaneously, this study develops the MMG algorithm to address the energy consumption balancing issue and establishes its efficacy *via* a large number of comparison tests.

## Supplemental Information

10.7717/peerj-cs.1118/supp-1 Supplemental Information 1 Raw dataset. Four datasets of the execution times in second for an image recognition task when executed in different machines/edge servers.

10.7717/peerj-cs.1118/supp-2 Supplemental Information 2 Implementation Code.

## Additional Information and Declarations

### Data Availability

The following information was supplied regarding data availability: The raw data and code are available in the Supplemental Files.
