# Supplementary material for: Blockchain based energy efficient multi-tasking optimistic scenario for mobile edge computing
Source: PeerJ Comput Sci. 2022 Oct 17;8:e1118. doi: 10.7717/peerj-cs.1118 (PMC9680867; doi:10.7717/peerj-cs.1118)
Supplement: Supplemental Information 1 — Four datasets of the execution times in second for an image recognition task when executed in different machines/edge servers. [file peerj-cs-08-1118-s001.zip › TATDescriptionDataset.zip!/TATDescriptionDataset/DatasetExpeirmentalDescription.pdf]

Execution Times for Image Recognition Task offloading in Mobile Edge Computing

This file contains four (4) datasets of the execution times in second for an image recognition task when executed in different machines/edge servers. They are being saved as .csv files. The edge servers used in the experiment are showing in the following figure:

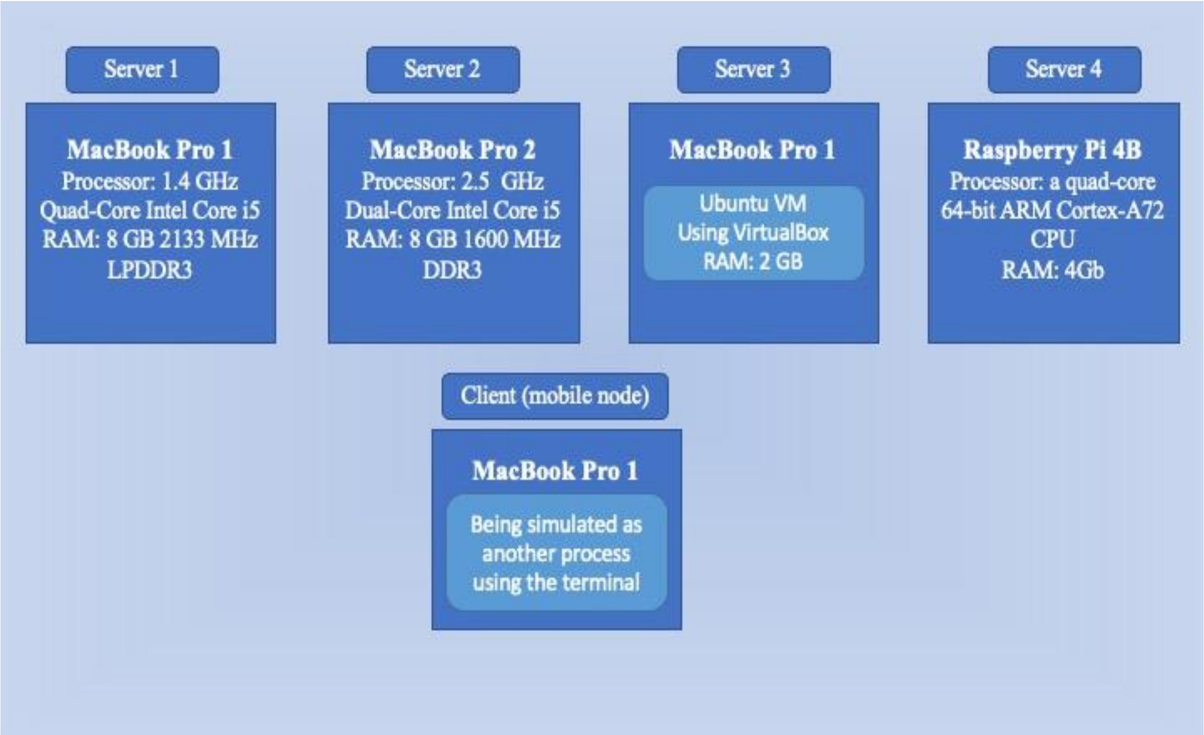

There are 4 files. Each csv file contains the execution times for each edge server. Each dataset contains two columns: local time and the actual execution time of the task.

During the experiment, at each second, the client (edge mobile node) sends an image to the edge server to process the task. The edge server performs the image recognition and returns the results of the prediction. A machine learning Python trained library (imageai.Prediction) has been used for this task. We calculated the *task execution time* per edge server, which is the time from *transferring* the image to the edge server until getting the image recognition result back to the mobile edge node, i.e., the turnaround time (TAT).
